# Prize Essay on the Zymotic Theory of Essential Fevers and Other Disordered Conditions of the Blood; Together with an Appendix on Medical Theories and Vital Statistics

**Published:** 1853-12

**Authors:** 


ART. VI.—*Prize Essay on the Zymotic Theory of Essential Fevers and other disordered conditions of the Blood; together with an Appendix on Medical Theories and Vital Statistics.* By SAMUEL S. ARMOUR, M. D., Cleveland, Ohio.

We owe an apology to Dr. Armour for our long delay in noticing this essay. It had been mislaid, and we have only now perused it. The intention of this essay is to support the theory of Zymotic Poisons. Dr. A., in accordance with this idea, considers the essential fevers as products of a changed condition in the blood. He does not, however, suppose a unity of cause or condition in the different fevers, and argues from the different symptoms presented, and from the different tissues affected, that the causes are distinct and unassociated. Thus small-pox attacks the dermoid tissues, while urea and its compounds affect the brain and nervous system, and are apt to give rise to low grades of inflammation in the serous and sero-fibrous tissues.

Our author then compares these local affinities to the localized action of calomel, ergot, and croton-oil. He then proceeds to prove, that actual and ascertainable changes in the blood do take place in these diseases, and quotes to some extent from chemical analyses to this effect.

Next follow some hypotheses as to specific contamination of blood structure. He supposes that some poisons affect different portions of the blood, some being comparatively innoxious, others *destroying* some element of the blood, so that it is never susceptible of a renewed action of the same poison; while others still, entering into chemical union with some parts of the blood essential to life, destroy its vitality, and produce necræmia, or death, beginning in the blood.

To our sense this is extremely fanciful. Were (as our author's hypothesis supposes) the poison of small-pox (e. g.) to destroy some element of the blood, it is hardly probable that such destruction of any one component part would be reconcilable with subsequent and entire good health. It is not supposable that the trivial poison of vaccination utterly destroys any one element of the blood; were this so, the not uncommon instances which occur, where the same individual has had vaccinia, mumps, varicella, scarlatina, and rubeola, would imply an enormous deficit in the elements of the vital fluid.

The Essay closes with brief remarks upon treatment, but as this involves no new idea, we omit to notice it, further than to say, that it is a sensible view of the different modes of cure, viz., first by counteracting the action of the poison; second, by expelling it from the system. S. B. H.

